# Draft Genome Sequence of *Lactobacillus reuteri* Strain CRL 1098, an Interesting Candidate for Functional Food Development

**DOI:** 10.1128/genomeA.00806-16

**Published:** 2016-08-25

**Authors:** Andrea C. Torres, Nadia E. Suárez, Graciela Font, Lucila Saavedra, María Pía Taranto

**Affiliations:** Centro de Referencia para Lactobacilos (CERELA-CONICET), Chacabuco, San Miguel de Tucumán, Tucumán, Argentina

## Abstract

We report here the draft genome sequence of *Lactobacillus reuteri* strain CRL 1098. This strain represents an interesting candidate for functional food development because of its proven probiotic properties. The draft genome sequence is composed of 1,969,471 bp assembled into 45 contigs and an average G+C content of 38.8%.

## GENOME ANNOUNCEMENT

*Lactobacillus reuteri* CRL 1098, a lactic acid bacterium isolated from sourdough, has gained attention for the development of functional foods as a potential probiotic. This strain is a well-known cobalamin producer strain (1). Furthermore, Malpeli et al. (2) demonstrated the hypocholesterolemic effect associated with the daily consumption of a yogurt containing *L. reuteri* CRL 1098.

To further investigate the probiotic potential of *L. reuteri* CRL 1098, we have determined its genome sequence. Genomic DNA was isolated by the method of Pospiech and Neumann (3) and used to generate reads sequences by a whole-genome shotgun (WGS) strategy on an Illumina MiSeq sequencer. Quality-filtered reads were *de novo* assembled into 45 contigs using the NGen DNAStar (versus 12.2.0) assembler (MR DNA, Shallowater, TX).

Genome annotation was done by the NCBI Prokaryotic Genome Annotation Pipeline (PGAP) service and the Rapid Annotations using Subsystems Technology (RAST) server (4).

The draft genome of *L. reuteri* CRL 1098 is 1,969,471 bp in length and has an average G+C content of 38.8%. A total of 1,968 coding sequences (CDSs) and 85 structural RNAs were predicted. Among all CDSs, 1,448 (73.6%) were assigned to known protein functions, while 520 (26.4%) remain as hypothetical proteins. Additionally, there are 303 RAST subsystems represented in the chromosome, which represent only 48% of the sequences assigned.

*In silico* studies revealed that *L. reuteri* CRL 1098 carries 29 genes involved in the cobalamin biosynthesis and two components of the cobalamin transporter in the *cbi-cob-hem* cluster.

Detailed analysis of the *L. reuteri* CRL 1098 genome will be useful to predict the competitiveness of the strain as a probiotic.

### Accession number(s).

This whole-genome shotgun project has been deposited at DDBJ/ENA/GenBank under the accession no. LYWI00000000. The version described in this paper is version LYWI01000000.
